# Clinical observation of the regeneration process of defects after breast cancer resection

**DOI:** 10.1186/s12905-021-01219-2

**Published:** 2021-03-06

**Authors:** Jun-jie Li, Ye Yang, Qi Wan, Hui Li, Qi-ming Long, Pu-rong Zhang

**Affiliations:** 1 Department of Breast Surgery, Sichuan Cancer Hospital, South Renmin Road Chengdu, No. 55, Section 4, Chengdu, 610041 Sichuan China; 2 Department of Medical Oncology, Sichuan Cancer Hospital, Chengdu, 610041 Sichuan China; 3 Jinjiang Maternity and Child Health Hospital, Chengdu, 610065 Sichuan China

**Keywords:** Breast cancer, Breast conserving surgery, Oxidized regenerated cellulose, Gelatin sponge, Defects repair

## Abstract

**Background:**

The present study aims to use two different kinds of filling materials, oxidized regenerated cellulose and gelatin sponge, to repair defects of breast-conserving surgery due to breast cancer, and compare the clinical efficacy, cosmetic effect and complication rate among groups.

**Methods:**

A total of 125 patients, who had breast -conserving surgery due to breast cancer, were enrolled into the present study. Postoperative efficacy was assessed by a doctor and patient, according to the Harvard/NSABP/RTOG Breast Cosmetic Grading Scale.

**Results:**

Among these patients, 41 patients received conventional breast-conserving surgery, and 84 patients received breast-conserving surgery plus filling implantation (41 patients in the oxidized regenerated cellulose group and 43 patients in the gelatin sponge group). All patients had small to medium sized breasts (cup size A and B). The average weight of tumor tissues was 56.61 ± 11.57 g in the conventional breast-conserving surgery group, 58.41 ± 8.53 g in the oxidized regenerated cellulose group, and 58.77 ± 9.90 g in the gelatin sponge group. The difference in pathological factors, average operation time, length of stay and local infection rate was not statistically significant among the three groups. 18 patients in the oxidized regenerated cellulose group and 15 patients in the gelatin sponge group were evaluated to have a good cosmetic effect by the surgeon and patient, while 12 patients in the conventional breast-conserving surgery group were evaluated to be have good cosmetic effect by the surgeon and patient. The cosmetic effects in the oxidized regenerated cellulose group and gelatin sponge group were comparable, and these were superior to those in the conventional breast-conserving surgery group.

**Conclusion:**

The use of oxidized regenerated cellulose and gelatin sponge is a feasible approach for defect repair after breast-conserving surgery.

## Background

The incidence of breast cancer has been increasing since the late 1970s [1]. Studies have shown that the incidence of breast cancer in women is 12.5% in the United States [1]. In recent years, the incidence of breast cancer in China has gradually increased [2]. Surgery is the first choice of treatment for non-advanced breast cancer [3]. For breast cancer patients with a large breast volume, the rotation of autologous tissue flaps can achieve a good cosmetic effect [4]. However, after conventional breast-conserving surgery for breast cancer patients with small and medium sized breasts, the lack of tissue repair often forms depression defects, which seriously affect the appearance of the breast [5–10].

For breast-conserving surgery, the primary goal is to ensure the negative margins of the residual cavity, and reduce its local recurrence [11–14]. In the case of small volume breasts, when the defect of the breast is too large to be closed and the remaining breast tissue is not enough to repair the residual cavity after the operation, this would still be insufficient for alternative surgical repairing techniques [15–20]. Furthermore, although breast prosthesis implantation technology has become very mature at present, this is only suitable for repairs after total mastectomy. It has been proven that breast prosthesis is not suitable for partial tissue defects after breast-conserving surgery. Vicryl mesh and oxidized regenerated cellulose are being developed for the repair of breast defects [21–24]. These materials temporarily fill these defects to prevent skin depression, and eventually, these would be absorbed by the body. However, compared with conventional breast-conserving surgery, Vicryl mesh implantation leads to more complications, while oxidized regenerated cellulose is a good filling material that can be used for partial breast defects without serious complications [25–27].

There are presently no comparative studies on oxidized regenerated cellulose and gelatin sponge for the repair of breast defects. Therefore, the present study aims to investigate the effect of the above artificial filling materials in the repair of local breast tissue defects after breast-conserving surgery.

## Methods

### Patients

A total of 125 breast cancer patients with small and medium sized breasts, who received breast-conserving surgery in the Breast Surgery Center of Sichuan Tumor Hospital from December 2016 to March 2019, were enrolled in this prospective study. The present study excluded patients with diabetic or mastitis, with poor blood glucose control (blood glucose level is not well-controlled by taking drugs and/or controlling diet, thus remains high). The present study was approved by the Ethics Committee of our hospital, and all patients provided a signed informed consent.

All enrolled patients were divided into two groups: conventional breast-conserving surgery group and artificial material implantation group. According to the type of filling material, patients in the artificial material implantation group were further divided into two groups: oxidized regenerated cellulose group and gelatin sponge group. The clinical factors that were evaluated included age, body mass index, resected breast tissue weight, tumor size, surgical margin status, operative time, length of hospital stay, and acute and chronic complications. The clinicopathological factors included tumor stage, hormone receptor status and adjuvant therapy. Postoperative adjuvant chemotherapy, and endocrine and targeted therapy were performed according to the patient’s condition, and all patients were treated with adjuvant radiotherapy. The Harvard/NSABP/RTOG Breast Cosmetic Grading Scale was used to evaluate the cosmetic effect of patients who had surgery for more than six months, including the surgeon’s evaluation and the patient’s self-evaluation [23].

### Study design

Raw material of oxidized regenerated cellulose is the cellulose made from original pulp of the German pine, which is then oxidized by nitrogen dioxide to form cellulosic acid, which, like other biologic physical materials, can be used as a platform for platelet aggregation and hemostasis. It contained about 19.5% carboxyl. It has many forms, such as mesh, gauze, fibrillar tufts, and sponges, all of which are absorbable and can be completely degraded within 1–6 weeks. The one used in this study was hemostatic gauze. Gelatin sponge is made of gelatin and does not contain biological activities. It does not have the helical structures of collagen, but it has hemostasis effects due to its porous structure. Oxidized regenerated cellulose and gelatin sponge used in this study were commercial products purchased from Ethicon, Inc., Johnson & Johnson Corporation in the United States and Jinling Pharmaceutical Co., Ltd in China, respectively.

The location of the tumor was confirmed by clinical physical examination and imaging examination before the operation, and the scope of tumor resection was determined. Intraoperative frozen pathological examination (internal, external, upper, lower and basal incisal margin) was performed to evaluate the surgical margins. If the margin was positive, the extended resection was performed again, and if the frozen pathological examination remained positive after the extended resection, a modified total mastectomy was performed. The position of the incisal margins was marked using a titanium clip. When the local postoperative defect failed to be repaired during the operation, it means that a depression deformity could form in the long term. Artificial material filling materials were used for patients in the artificial material implantation group, which were implanted with a cotton shape, and the shape was identical to the residual cavity to an appropriate degree. After the implantation of artificial materials, the incision was sutured layer by layer to maintain the appropriate tension of the skin. No drainage tube was inserted after the operation, in order to reduce the probability of infection. An appropriate amount of seroma can play a role of long-term organization and shaping. However, it should be noted that when the tension of the local seroma becomes too large after the operation, a syringe can be used for proper suction and decompression. Radiotherapy IMRT DT 50 Gy/25F/5W, axillary lymph node negative, radiotherapy site breast; Axillary lymph node positive breast and axillary. Clinical characteristics and pathological characteristics and staging among the three groups, absorption time between the two materials, as well as complications and survey results in the satisfaction of patients among the three groups were compared.

### Statistical analysis

The SPSS 19.0 software package was used for data analysis. The measurement data was presented as mean ± standard deviation. *X*^2^-test, One Way ANOVA and Kruskal–Wallis test was used for the statistical analysis of measurement data. *P* < 0.05 was considered statistically significant.

## Results

### Comparison of clinical characteristics of patients among the three groups

As shown in Table 1, the difference in mean age and body mass index was not significant among the three groups. The results revealed that three patients in the conventional breast-conserving surgery group, five patients in the oxidized regenerated cellulose group, and three patients in the gelatin sponge group had high blood pressure, but all patients were ideally controlled. The average weight of tumor tissues was 56.61 ± 11.57 g in the conventional breast-conserving surgery group, 58.41 ± 8.53 g in the oxidized regenerated cellulose group, and 58.77 ± 9.90 g in the gelatin sponge group. The difference in size of resected tumor tissue was not statistically significant among the three groups.

**Table 1:** Clinical characteristics of patients

	Breast-conserving surgery group (n = 41)	Oxidized regenerated cellulose group (n = 41)	Gelatin sponge group (n = 43)	*P*
Mean age (years, ± SD)	45.44 ± 8.97	44.41 ± 9.05	43.65 ± 7.94	0.639
Body mass index (kg/m^2^, ± SD)	2.22 ± 0.19	2.16 ± 0.18	2.19 ± 0.21	0.426
Resected tumor tissue (g, ± SD)	56.61 ± 11.57	58.41 ± 8.53	58.77 ± 9.90	0.690
Tumor size (cm, ± SD)	1.94 ± 0.48	1.76 ± 0.34	1.92 ± 0.47	0.103
Time of operation (minutes, ± SD)	149.66 ± 14.92	152.17 ± 15.14	152.30 ± 16.36	0.683
Time in hospital (days, ± SD)	13.98 ± 2.71	14.15 ± 2.65	14.30 ± 2.53	0.823

Age and operation time were normal distribution and homogeneity of variance, and one-way ANOVA was used. BMI, resected tissue, tumor size, and length of hospital stay were measured by the Kruskal–Wallis test

In the conventional breast-conserving surgery group, the surgical margins of five patients were positive in the first frozen section. In the oxidized regenerated cellulose group, the surgical margins of three patients were positive in the first frozen section. In the gelatin sponge group, the surgical margins of two patients were positive in the first frozen section, but the incisal margins turned to negative after the resection was enlarged again. The difference in average operation time was not statistically significant among the conventional breast-conserving surgery group, oxidized regenerated cellulose group, and gelatin sponge group, and the difference in infection rate was not significant among the three groups after the operation and radiotherapy.

### Comparison of pathological characteristics and staging among the three groups

As shown in Table 2, the difference in pathological features was not statistically significant among the three groups, which included tumor pathological type, T/N stage, total stage, estrogen receptor, progesterone receptor, HER-2 receptor status and adjuvant therapy. All patients with breast conservation received radiotherapy. No obvious complications were found in the artificial material implantation group.

**Table 2:** Pathological features and stages of the patients

	Breast-conserving surgery group (n = 41)	Oxidized regenerated cellulose group (n = 41)	Gelatin sponge group (n = 43)	*P*
Pathological pattern (n)				0.644
Invasive ductal carcinoma	38	36	40	
Ductal carcinoma in situ	3	5	3	
Tumor size (n)				0.916
Tis	3	5	3	
T1	29	28	30	
T2	9	8	10	
Lymph node status (n)				0.866
N0	35	36	36	
N1	6	5	7	
Staging (n)				
0	3	5	3	0.883
1A	26	25	28	
2A	8	9	7	
2B	4	2	5	
ER status (n)				0.533
Positive	26	30	27	
Negative	15	11	16	
PR status (n)				0.736
Positive	25	22	23	
Negative	16	19	20	
HER-2 status (n)				0.752
Positive	8	5	10	
Negative	33	36	33	
Triple negative breast cancer (n)	8	7	9	
Adjuvant chemotherapy (n)				0.877
Yes	30	28	31	
No	11	13	12	
Endocrine therapy (n)				
Yes	29	34	32	0.415
No	12	7	11	

Pearson chi-square test was used

### Comparison of absorption time between the two materials

There was a certain difference in absorption time between the two materials. Residual fibrosis gradually formed after one month in the oxidized regenerated cellulose group and gelatin sponge group. However, the gelatin sponge was absorbed more than the oxidized regenerated cellulose. Furthermore, the gelatin sponge was almost absorbed within nearly one month, while it took more than one month for the oxidized regenerated cellulose to be completely absorbed.

### Comparison of complications and survey results in the satisfaction of patients among the three groups

As shown in Tables 3 and 4, according to the cosmetic effect evaluated using the Harvard/NSABP/RTOG Breast Cosmetic Grading Scale, 18 patients in the oxidized regenerated cellulose group and 15 patients in the gelatin sponge group were evaluated to have a good cosmetic effect by the surgeon and patient, while 12 patients in the conventional breast-conserving surgery group were evaluated to be have good cosmetic effect by the surgeon and patient.

**Table 3:** Satisfaction survey results

	Patient	Excellent	Good	Fair	Poor
	Surgeon				
Traditional breast conserving surgery (n = 41)	Excellent	12	2	–	–
	Good	2	4	–	–
	Fair	–	2	5	3
	Poor	–	–	5	6
Oxidized regenerated cellulose (n = 41)	Excellent	18	3	–	–
	Good	7	5	–	–
	Fair	–	3	–	–
	Poor	–	–	1	4
Gelatin sponge (n = 43)	Excellent	15	8	–	–
	Good	8	3	–	–
	Fair	1	2	1	–
	Poor	–	–	2	3

TBCS, traditional breast conserving surgery; ORC, oxidized regenerated cellulose; GS, gelatin sponge

Satisfaction analysis: TBCS *VS* ORC (*P* = 0.024); TBCS *VS* GS (*P* = 0.038); ORC VS GS (*P* = 0.973) (Evaluation by surgeons). TBCS *VS* ORC (*P* = 0.005); TBCS *VS* GS (*P* = 0.014); ORC VS GS (*P* = 0.73) (Evaluation by patients)

**Table 4:** Complications in each group

Complication	Breast-conserving surgery group (n = 41)	Oxidized regenerated cellulose group (n = 41)	Gelatin sponge group (n = 43)
Rubefaction (n)	4	7	5
Wound infection (n)	2	4	3
Complication after radiotherapy (n)	3	5	4

## Discussion

In order to identify filling materials with a better effect and less side effects, the clinical and cosmetic effects between gelatin sponge and oxidized regenerated cellulose were compared, and the application in this field was explored. Oxidized regenerated cellulose and gelatin sponge are the commonly used artificial hemostatic fillers in clinic. Using these to fill the residual cavity after breast-conserving surgery does not increase the operation time and the incidence of complications [5, 6, 22, 23]. Therefore, it was speculated that these two materials may play a role in the repair and shaping of the breast, while achieving the goal of hemostasis. Furthermore, these present results also revealed that the cosmetic effect of these two materials was better, when compared to the conventional breast-conserving surgery group.

However, in the present study, it was not observed that the infection rate in the artificial material implantation group was higher than that in the conventional breast-conserving surgery group. However, according to the experience of the investigators, artificial materials cannot have direct contact with the dermis after implantation. Otherwise, this will increase the local rejection. This rejection can result in redness of the local skin for approximately a week or so. The two different types of artificial materials used in the present study are commonly used as clinical artificial hemostatic fillers.

A number of conditions should be met by filling materials for local defects after breast cancer surgery. First, the material should be able to maintain its availability until the defect forms fibrosis, in order to achieve good cosmetic results. Second, postoperative changes should be clearly distinguished from the real recurrent lesion area of breast cancer. Third, the material should be capable of withstanding radiotherapy or chemotherapy, without serious complications [23–27]. The absorption time of oxidized regenerated cellulose is slightly longer than that of gelatin sponge. Furthermore, the filling time of subsequent fiber cells is longer, and the amount of tissue filling is larger, which ensures the beauty of the appearance. However, the growth of too many fiber cells affects a certain sense of touch. Although the filling amount of fiber cells is slightly lower in the gelatin sponge group, when compared to the former, the local softness is better.

The feasibility of using oxidized regenerated cellulose as filling materials for partial breast defects has been reported in foreign countries. In the present study, the gelatin sponge with a similar clinical effect also achieved good results. The present study further confirms that oxidized regenerated cellulose is suitable for filling partial breast defects, and that gelatin sponge is also a feasible choice. However, there is no reference to possible positions in which the implant and its properties would be described, which need further exploration.

Limitation of this study is that the sample size is not very large. In future studies, we will enroll more subjects to further prove our findings.

## Conclusion

The present study reveals that oxidized regenerated cellulose and gelatin sponge are feasible filling materials for partial breast defects. The difference in operation time and incidence of postoperative infection is not significant, when compared to the conventional breast-conserving surgery group, and the cosmetic effect is significantly better than that in the conventional breast-conserving surgery group. Oxidized regenerated cellulose and gelatin sponge have its own advantages in terms of cosmetic effect. According to the specific situation, the surgeon can choose these as filling materials for local breast defects after breast-conserving surgery.

## Data Availability

The datasets used and/or analysed during the current study available from the corresponding author on reasonable request.
